# OTO: Ontology Term Organizer

**DOI:** 10.1186/s12859-015-0488-1

**Published:** 2015-02-15

**Authors:** Fengqiong Huang, James A Macklin, Hong Cui, Heather A Cole, Lorena Endara

**Affiliations:** School of Information Resources and Library Science, University of Arizona, Tucson, USA; Agriculture Agri-Food Canada, Ottawa, Canada; Department of Biology, University of Florida, Gainesville, USA

**Keywords:** Biodiversity informatics, Controlled vocabularies, Web-based application, Consensus-promoting, Community software

## Abstract

**Background:**

The need to create controlled vocabularies such as ontologies for knowledge organization and access has been widely recognized in various domains. Despite the indispensable need of thorough domain knowledge in ontology construction, most software tools for ontology construction are designed for knowledge engineers and not for domain experts to use. The differences in the opinions of different domain experts and in the terminology usages in source literature are rarely addressed by existing software.

**Methods:**

OTO software was developed based on the Agile principles. Through iterations of software release and user feedback, new features are added and existing features modified to make the tool more intuitive and efficient to use for small and large data sets. The software is open source and built in Java.

**Results:**

Ontology Term Organizer (OTO; http://biosemantics.arizona.edu/OTO/) is a user-friendly, web-based, consensus-promoting, open source application for organizing domain terms by dragging and dropping terms to appropriate locations. The application is designed for users with specific domain knowledge such as biology but not in-depth ontology construction skills. Specifically OTO can be used to establish is_a, part_of, synonym, and order relationships among terms in any domain that reflects the terminology usage in source literature and based on multiple experts’ opinions. The organized terms may be fed into formal ontologies to boost their coverage. All datasets organized on OTO are publicly available.

**Conclusion:**

OTO has been used to organize the terms extracted from thirty volumes of Flora of North America and Flora of China combined, in addition to some smaller datasets of different taxon groups. User feedback indicates that the tool is efficient and user friendly. Being open source software, the application can be modified to fit varied term organization needs for different domains.

## Background

The Ontology Term Organizer (OTO) tool was initially developed to facilitate consensus-based categorization of the terms used to describe the morphology or phenotype of organisms. The description of organisms new to science, and the continuous process of revising and improving them based on new evidence, is traditionally done by taxonomists and systematists. The publications that they produce contain descriptions that are typically highly detailed and contain terms which range from general to highly specific in their usages. When compared across resources, these terms can vary from identical to similar to non-overlapping within and between major groups of organisms. This lack of consistency presented a serious challenge to the authors when attempting to extract knowledge from legacy descriptive taxonomic literature as part of a National Science Foundation-funded project entitled: “Fine-Grained Semantic Markup of Descriptive Data for Knowledge Applications in Biodiversity Domains”.

Morphological descriptions are often composed in a telegrammatic style, for example:

Leaves alternate, spirally arranged, 2--3-ranked, simple; stipules deciduous, distinct; petioles present. Leaf blade sometimes lobed, pinnately veined, margins toothed, serrate to nearly entire; surfaces glabrous to tomentose, abaxially often with resinous glands. [Betulaceae, v.3. Flora of North America North of Mexico [1].

The description provides detailed information about the taxon, but knowledge beyond the text is required to fully understand the descriptions as it does not explicitly state that *leaf* is a plant organ (leaf is_a organ), that *alternate* is a way the leaves are arranged (alternate is_a arrangement), that *stipules* and *petioles* are part of the leaves (stipule part_of some leaf, petiole part_of some leaf), or what intermediate states between *serrate* and *nearly entire* for margin shape or between *glabrous* and *tomentose* for pubescence or hairiness are possible. To enable intelligent organization and use of organism-based morphological information (e.g., to generate phylogenetic matrices, to support machine reasoning, or to compare taxon morphological profiles), the detailed semantics need to be made explicit for computers to use. Among those the most fundamental semantics that need to be defined for the terms include is_a and part_of relationships as illustrated above, in addition, it is valuable to link synonyms and to define the semantic distance for the states that fall in a natural order. The latter will help the computer to reason that *toothed leaves* are more similar to *serrate leaves* than *entire leaves*.

To pin-down the semantics of domain terms, categorical glossaries and ontologies have been constructed (e.g. [2-6]), but they lack agreement. An evaluation of four glossaries/ontologies relevant to botany found that agreement was less than 50% [7].

Past research has shown that different taxon groups employ different vocabularies and new terminology are constantly encountered [8]. In a recent experiment, a 2013 version of the Uberon ontology (a cross-taxon anatomy ontology, [9]), PATO [5], and BSPO [10] were used to annotate a set of 203 character descriptions taken from phylogenetic matrices. As much as 35% of the unique terms needed for the annotation were not found in the ontologies (manuscript in preparation). UMLS Metathesaurus [11] is arguably the most complete thesaurus/ontology for medicine and has been in active development for at least half of a century (one of its component MESH was introduced in 1963). A recent evaluation by Friedlin and Overhage [12] of the UMLS Metathesaurus found “a large portion of concepts [>70%] found in clinical narrative documents (admission, discharge, and chest x-rays) are either unrepresented [3-4%] or poorly represented [>66%] in the current version of the UMLS Metathesaurus”. The mismatch of terms used in the documents and those included in the thesaurus due to various form transformations have been cited as the main difficulty in using this and other large scale controlled vocabularies (e.g. Library of Congress Subject Headings [13]). These controlled vocabulary evaluations suggest that, while the coverage of the ontologies are constantly improving, the vocabulary control is a long term, continuous process. Care needs to be taken in transforming the terms for a controlled vocabulary and to provide a useful path to link the terms used in the literature to the transformed terms included in the controlled vocabularies.

Another factor that hinders the vocabulary control process is the set of diverse knowledge and skills required to perform the task. Individuals with rich knowledge in a subject domain for example biology are often not the individuals with the skills needed for ontology construction, yet both knowledge and skills are needed to perform the task of vocabulary control. Thus, knowledge modeling tools friendly to domain experts are urgently needed [14].

Taking the above issues into consideration, we developed the Ontology Terms Organizer (OTO; http://biosemantics.arizona.edu/OTO/) to assist a domain expert in organizing sets of terms extracted from their source literature with is_a, part_of and/or order semantics. The organized terms can be used by knowledge engineers and integrated into domain glossaries, thesauri, or ontologies. OTO is, to our knowledge, the first consensus-promoting, usage-informed, drag-and-drop based, online term organization tool designed for use by biologists or other domain experts who have rich domain knowledge but are not equipped to deal with the intricacy of formal knowledge representation using tools such as Protégé [15], TopBraid [16], and several dozen of other ontology editors (e.g. see Wikipedia page on “ontology editor”). OTO is not an ontology editor, but a tool that bridges the knowledge of domain experts and knowledge engineering.

Although OTO was initially created for biologists to categorize anatomical and morphological terms found in biodiversity literature, it can also be used generically to organize terms for any other domain, where is_a, part_of, and/or order relationships are required.

## Implementation

**Figure 1:**
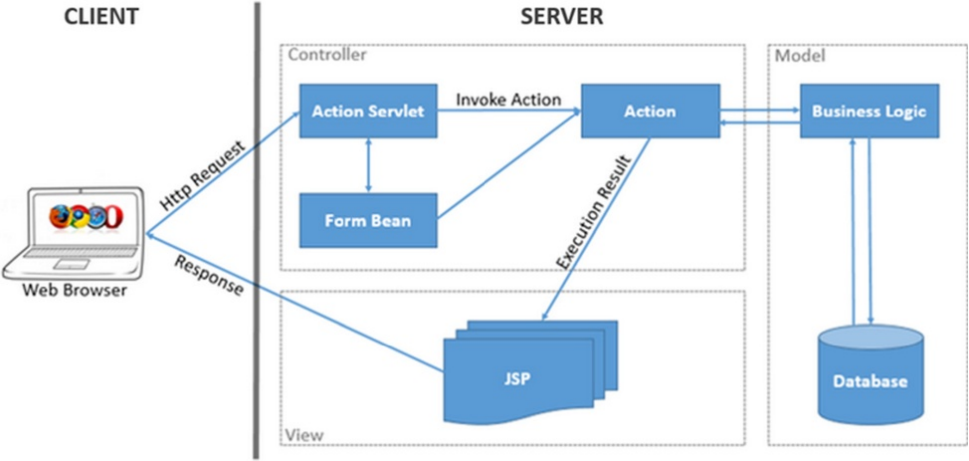
**OTO system architecture.** OTO employs client-server architecture and utilizes the Model-View-Controller design pattern. Web Browser as the client sends requests to the Controller, which accesses the Model and executes the requests. Execution results are presented as views and sent back to Web Browser.

Figure 1 shows the system architecture of OTO. OTO uses the popular Apache Struts web framework [17] and Model-View-Controller [18] design pattern. The implementation of OTO relies on standard technologies such as Java Beans, Java Servlets, Java Server Pages (JSP) and XML (eXtensible Markup Language).

**Figure 2:**
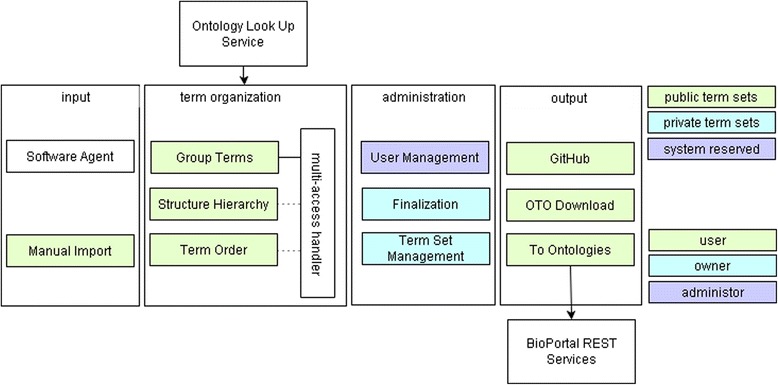
**OTO functional components.** OTO supports a set of input, term organization, administration, and output functions. It defines three user roles: user, owner, and administrator and three dataset types: public, private, and system reserved. The role of a user determinates the user’s privileges on functions and datasets. OTO also utilizes two web services: Ontology Look up Service and BioPortal REST Services.

Function-wise OTO includes input, term organization, administration, and output components, as shown in Figure 2. Sets of terms to be organized (called term sets or datasets) are loaded into OTO either via software such as CharaParser^a^ [19] or through manual import. Term organization functions are provided for one or multiple users, including: (1) Group Terms for organizing is_a relationships and synonym relationships, (2) Structure Hierarchy for organizing part_of relationships, and (3) Term Order for organizing order relationships. When a term set is considered organized using one or more of the functions, user’s decisions are then approved, or finalized, and an output is generated. OTO can output: (1) csv files pushed to GitHub (https://github.com/biosemantics/glossaries), (2) zipped files downloadable via the OTO website, and/or (3) term requests sent to ontologies hosted at BioPortal [20].

OTO also uses several web services to support its functionality. In OTO, a term is always accompanied by the source sentences where the term was found and a set of possible definitions provided for the term by other controlled vocabularies. For example, OTO uses the web services provided by the Ontology Lookup Service [21] to retrieve the definition of a term from the Phenotype Quality Ontology (PATO). Such information is available to the users in OTO to facilitate their decision-making process. OTO also allows a user to submit a term to a selection of existing ontologies in BioPortal and BioPortal REST Services [22] are employed for this purpose.

OTO delegates version control function to Github [23]. When a term set is organized and approved by the responsible user, OTO will generate a version number for the term set, commit and push it to the biosemantics/glossaries repository on Github.

Users in OTO have different roles that are associated with privileges (Figure 2). The three roles OTO differentiates are users, term set owners, and administrators:

1. A user is anyone registered on the OTO website. A user is granted access to all the public datasets once her/his registration is approved by an administrator. A user can organize terms in any public term set, send term requests to the existing ontologies, and access finalized datasets either on OTO/via Github, as finalized term sets are publicly accessible.
2. A term set owner is the user who creates/uploads a term set. Every term set has an owner. In addition to the privileges of a user, a term set owner can dedicate the public/private status of an owned term set, delete an owned term set, merge owned datasets, review user term organization decisions, and finalize/reopen an owned term set.
3. Administrators of OTO are responsible for user management (i.e., approve/revoke a user) and have full privileges over all public and private datasets, including system reserved datasets.

A term set in OTO can be private, public, or system reserved. While private datasets are organized by the owner only and managed by the owner and administrators, any user can organize the terms in a public or a system reserved term set, and only an administrator can manage the system reserved term set (Figure 2). OTO currently holds five system reserved datasets, one for each of plants, hymenoptera, algae, porifera and fossil groups, that have already been reviewed by domain experts. System reserved datasets have a fixed naming format of *Type*_glossary in OTO, e.g. Plant_glossary, and is marked as “[System Reserved].” One goal of OTO is to progressively grow the system reserved glossaries from smaller datasets of the same group. When a term set (private, public, or system reserved) is finalized, it becomes accessible for all.

A MySQL database server is used to store all the data OTO uses or generates. As OTO holds multiple independent datasets, there are a set of tables that hold general data to support the general functionality of OTO, as well as a set of tables for each term set.

## Results and discussion

In this section we describe OTO functionalities from the user’s perspective. Readers who are interested in learning OTO functionalities can log onto OTO with username *OTOdemo* and password *OTOdemopass* and work on the OTO_Demo dataset, which is one of many datasets currently hosted on OTO.

### Input functionality

**Figure 3:**
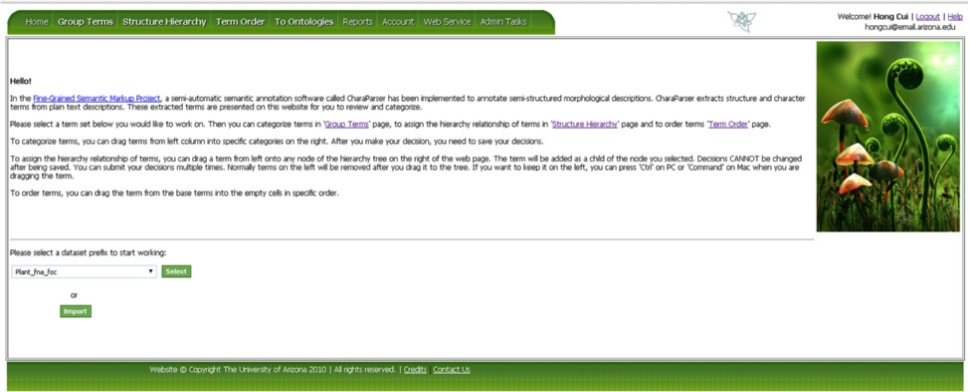
**OTO Welcome page.** User can select an existing dataset to organize, or import a new dataset.

**Figure 4:**
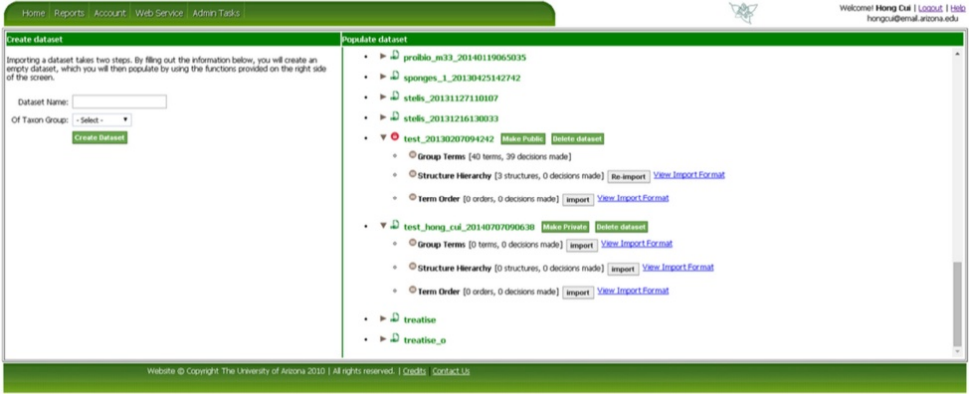
**Import datasets.** To import a dataset, first create an empty dataset (Left), then populate the newly created dataset by clicking on the import/Re-import button (Right).

As mentioned before, OTO can take an input dataset from CharaParser [19], which is a text mining system developed to parse organism morphological descriptions. CharaParser also extracts domain terms from the descriptions and uploads these terms automatically to OTO. Generally, datasets meeting OTO format requirements can be manually imported into OTO. Figure 3 shows the “Welcome” page of OTO after login where the user can invoke the Import function. Figure 4 is a screenshot of the data import page in OTO. A *dataset* could have one to three *term sets* for Group Terms, Structure Hierarchy, and/or Term Order tasks. The owner of a dataset can set the public/private status for the dataset and import/re-import data for different term organization tasks. Only the term sets that have not started being organized can be re-imported. Besides the name for a dataset, the taxon group the terms describe also needs to be specified when importing a term set. Taxon group information is needed for certain term set management tasks such as merging datasets.

### Term organization functionality

As OTO aims to promote consensus-based controlled vocabulary building, the most important design goal of OTO is to provide a user-friendly interface for them to express their opinions on how terms should be organized. OTO provides three ways to organize terms using drag-and-drop functionality. The first is term categorization that involves assigning one or more categories to a term, which is equivalent to assigning is_a relationships. This can be done on the *Group Terms* page of OTO. The second is to sort out part_of relationships among terms representing some entities, such as organs and their parts. The part_of relationships are rendered in a tree structure on the *Structure Hierarchy* page. The third term organization page, *Term Order*, serves to put range-valued categorical descriptors (e.g., terms describing varied levels of hairiness) in order. OTO records the order of terms in that group.

#### Group terms

**Figure 5:**
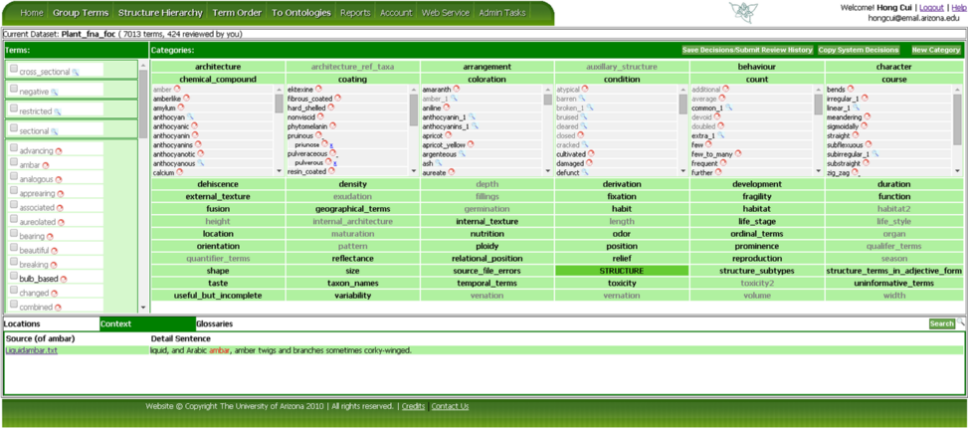
**OTO Group Terms page.** Terms shown in the left pane are to be categorized (drag and drop) into the categories shown in the right pane. More information about a selected term can be found in the lower pane, which consists of three tabs: Location, Context, and Glossary.

Figure 5 shows the Group Terms page in OTO. On the top of the page, the dataset name and the user’s progress are shown. The terms to be grouped/categorized are shown in the left column and the categories are shown to the right. A set of default categories the dataset owner imported are shown initially. The user can add new categories by clicking on “New Category.” Multiple terms can be selected by clicking on the check boxes and dragging them all at once to a new category. Clicking on a term shows term related information in the Location, Context, and Glossaries panels displayed on the lower part of the screen. Location shows the current categories a term resides in. The Search button at the right side of the panel has a similar function but it allows the user to enter any term to search. Context shows the source sentences the term appears in (e.g., Figure 5 shows the source sentence for the term *amber* in the dataset Plant_fna_foc). Glossaries display the definitions of the term in existing controlled vocabularies, currently including the system reserved glossary and PATO. Collectively Location, Context, and Glossaries provide term related information to facilitate the term categorization process. Every hour, users are reminded to save their decisions. Terms that are not categorized (i.e. left in the Terms column) are not included in the final results when the term set is finalized.

It is common for a term to belong to multiple categories as they may be homonyms or have different meanings under different contexts. OTO allows the user to copy a term from one category to another by holding down the Ctrl key while dragging (or the Command key on MAC). Terms that are found in multiple categories are renamed with a numerical index (e.g., *sweet*_1, *sweet*_2). There are often synonyms in a term set. Synonym relationships can be established by dragging one term onto another term. Note the synonym relations can be established only after the categorization decisions have been saved for both terms involved. Synonymy relationship can be removed by clicking on the blue “x” next to the synonym. Figure 5 shows examples of synonyms in the “Coating” category and multi-category terms (denoted by the underscore and number) in the “Coloration” category. The multi-category and synonym features used together help to move a set of unassigned terms toward a state that is better controlled. When synonyms exist, the term best representing the concept should be considered the preferred term and other synonyms should be synonymized to the preferred term. Whenever possible, avoid using terms with multiple categories (i.e. terms with a numerical index) as the preferred term because they are ambiguous. The combination of these practices will result in a sound controlled vocabulary (one term represents one concept and vice versa) with the maximized searchability (variations used in the literature are synonymized and linked to the preferred term). It also increases the chance for natural language processing techniques to perform Word Sense Disambiguation successfully on the ambiguous terms.

**Figure 6:**
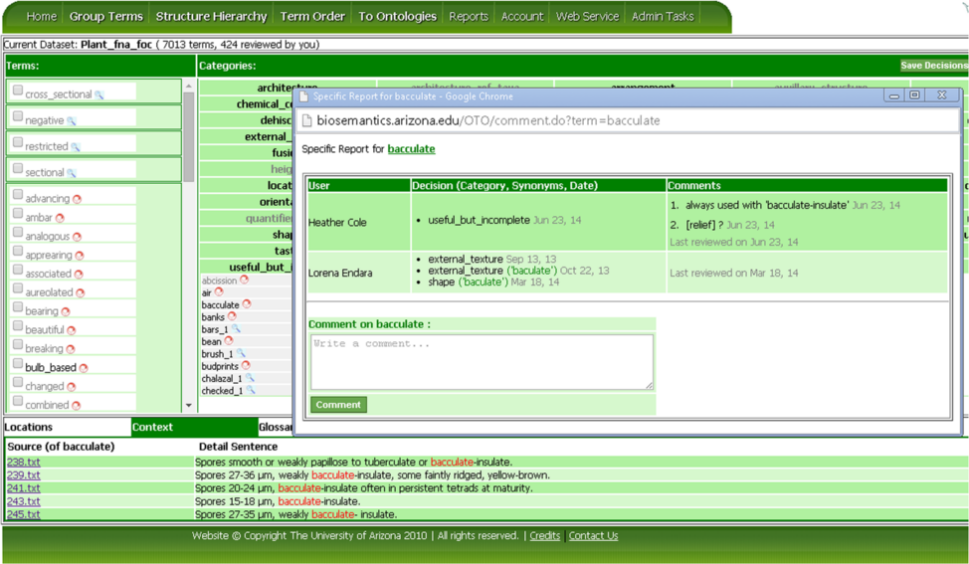
**Term context and term report in OTO.** Select a term and then click on the Context tab to show the original sentences the term appears (this works only when source sentences have been imported as part of the dataset). Click on the blue magnifying glass or the red circle icon next to a term to bring up the report holding the categorization history of the term. Shown in the figure is the context and term report for the term *bacculate*.

Each term on the Group Terms page is associated with a report, which includes the complete categorization history for the term. The report is opened when the user clicks on the blue magnifying glass or the red circle icon next to the term. The red circle indicates that different categorization decisions have been made by different users. Figure 6 shows a term report for the term *bacculate*. The report window provides a comment area, where the user can write down their thoughts, which remind the user and informs others why a particular decision was made.

Two other features that make the term categorization process more efficient include Copy System Decisions and automatic display of the most recent decisions. Copy System Decisions (Figure 5, top right corner) copies the categorization decisions from the system reserved glossaries of the same taxon group to the current term grouping task, or phrased differently, terms that have matches in the system reserved glossaries are automatically dropped into the corresponding categories. The greater the coverage of the system reserved glossaries, the less effort required of the user in categorizing the terms. After the categorizations are copied, however, the user can still override the system provided decisions with his/her own categorization. The automatic display of the most recent decisions from all users who have worked on a term set allows a later user to simply review or change previous decisions (made by others) to his/her satisfaction and not have to start from the beginning.

### Structure hierarchy

**Figure 7:**
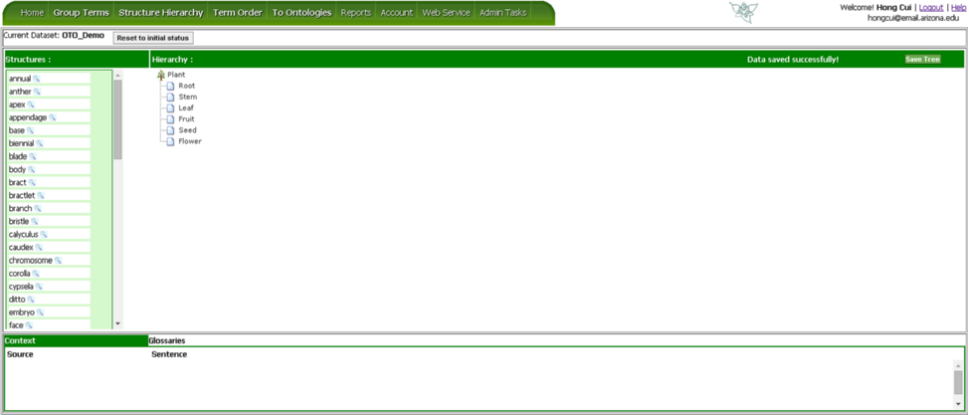
**OTO structure hierarchy page.** Terms shown in the left pane are to be inserted (drag and drop) into the hierarchy shown in the right pane. More information about a selected term can be found in the lower pane, which consists of three tabs: Location, Context, and Glossary.

**Figure 8:**
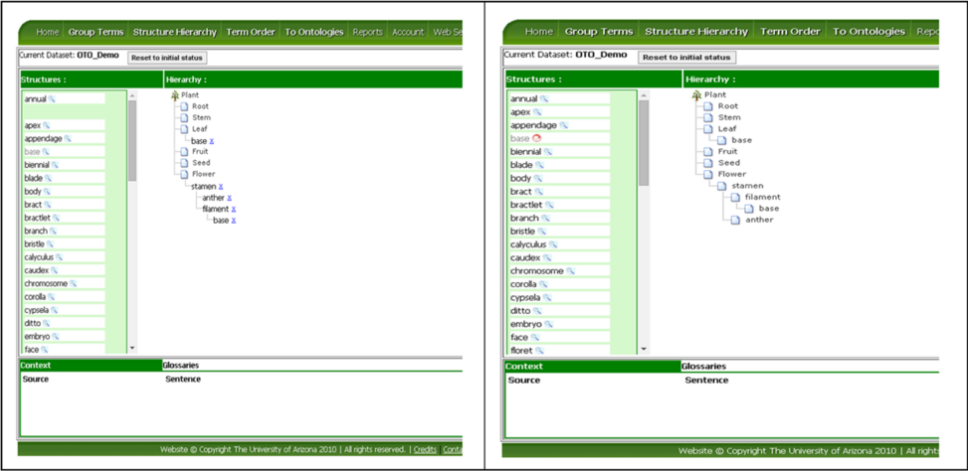
**Add nodes into the Hierarchy.** Left: Four nodes are just added to the bottom of the hierarchy. Right: After the hierarchy is saved, the new nodes become part of the hierarchy, conflicting decisions on the node *base* are detected, and a red circle icon is placed by the term in the left pane.

**Figure 9:**
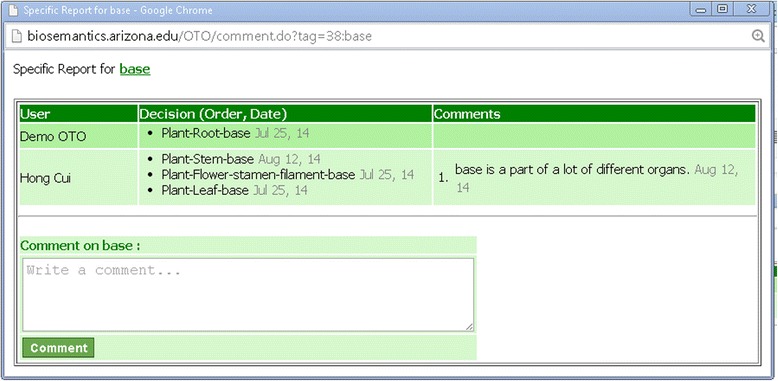
**Conflicting hierarchy decisions shown in the Term Report.** Three different decisions made by two users on the term *base* are shown.

The Structure Hierarchy page is used to associate entity terms via part_of relationships. The interface layout is similar to the Group Term page as the user drags terms from the left column (“Structures:”) to the Hierarchy canvas to the right. The Hierarchy canvas is initialized with a default part_of hierarchy, for example, Figure 7 shows the initial part_of hierarchy for Plant. The user can drag a term from the left and drop it on top of a node in the hierarchy to create a child node. Next to each of the un-saved nodes, there is an “x”, which can be clicked to remove a node (Figure 8). As illustrated in Figure 8, some structure terms are used once in the Hierarchy, for example *stamen* because stamens are part of the flower organ alone, while others may need to appear at different locations in the Hierarchy, for example *base*, as many structures may have a base. If the user holds down the Ctrl key while dragging/dropping a term, the term will remain in the term list (but turn grey) after being added to the Hierarchy so they can be reused. Similarly to the Group Term page, conflicting user decisions in constructing the Hierarchy are denoted with the red circles (Figure 8) and recorded in the Term Report (Figure 9).

### Term order

The Term Order page is designed to sort the categorical values of entity attributes that fall in some natural order. For example, by wave length, colors can be sorted into the order: Violet, Blue, Cyan, Green, Yellow, Orange, and Red. Such orders provide useful semantic information that otherwise would be missing for computer or human agents to determine, for example, that Blue is more similar to Cyan than to Yellow. In morphological descriptions, such ordered categorical values are used but typically without precise definition, for example, the stems of a plant may be described as “… usually erect, sometimes *prostrate to ascending*”. The goal of the Term Order page is to invite domain experts to help define these orders, hence enabling a more precise understanding of *prostrate to ascending* and what intermediate states may be between *prostrate* and *ascending*.

**Figure 10:**
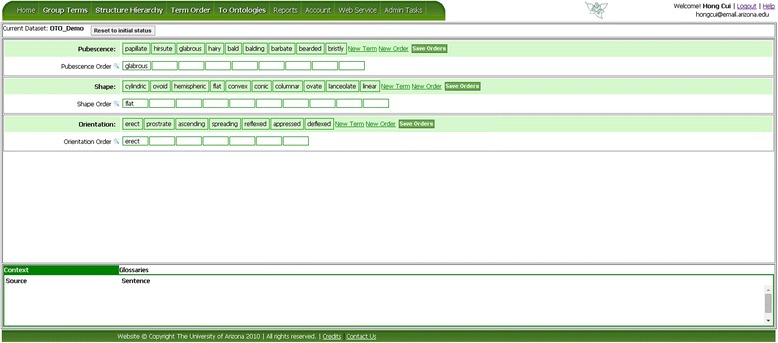
**OTO Term Order page.** The top pane shows three sets of terms to be ordered: Pubescence, Shape, and Orientation. In each set, the first row lists the terms to be rearranged (drag and drop) into an order in the subsequent row(s). More information about a selected term can be found in the lower pane, which consists of three tabs: Location, Context, and Glossary.

**Figure 11:**
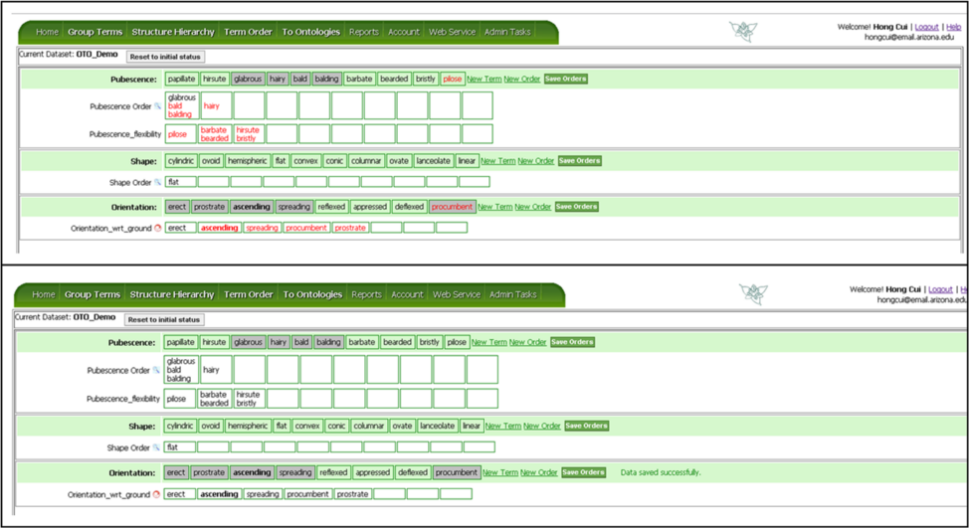
**Ordering term and save orders.** The terms shown in red are the unsaved edits in the orders. When the orders are saved, the terms turn black and become part of the orders.

Figure 10 shows the Term Order page when it is initially displayed. Here three sets of terms are to be ordered for pubescence, shape, and orientation. The user can add new terms to the term lists (New Term) and add new empty orders (New Order). The user can also edit the name of the orders by mousing over an order name and clicking on the pencil icon when it is shown. The latter two features are provided with the knowledge that a set of terms belonging to the same term categories may be ordered based on different criteria. For example, colors can be ordered based on wave length (or hue), but they can also be ordered by saturation or brightness. In our example (Figure 11), some terms in the orientation category can be ordered based on their orientation with respect to the ground, some terms w.r.t. the supporting structure or even to itself. The user can create multiple orders as needed to sort a group of terms.

**Figure 12:**
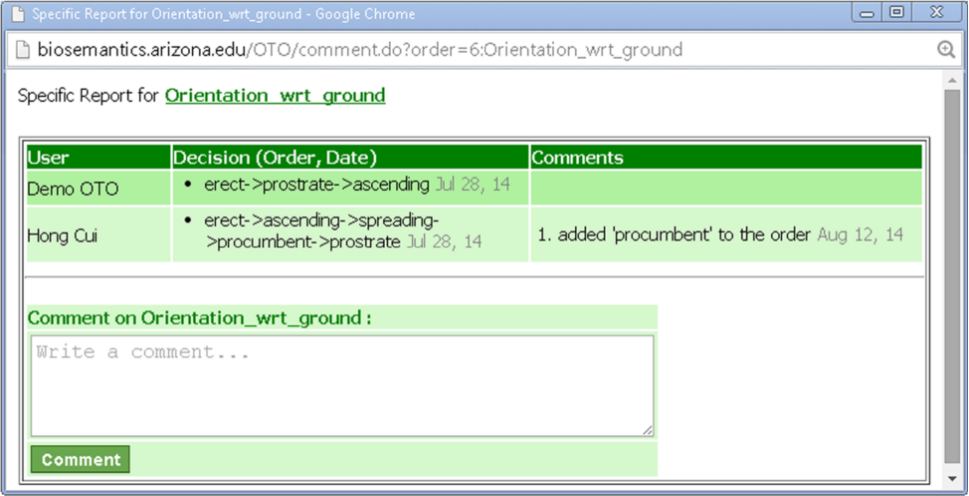
**Conflicting order decisions shown in the Term Report.** Two conflicting decisions are shown for the order Orientation_wrt_ground.

If several terms are deemed similar in a given context for an order, they can be dragged into the same box (Figure 11). Similar to other term organization pages, conflicting decisions are signaled with red circles and displayed in term reports (Figures 11 and 12).

### Dataset finalization and management functionalities

**Figure 13:**
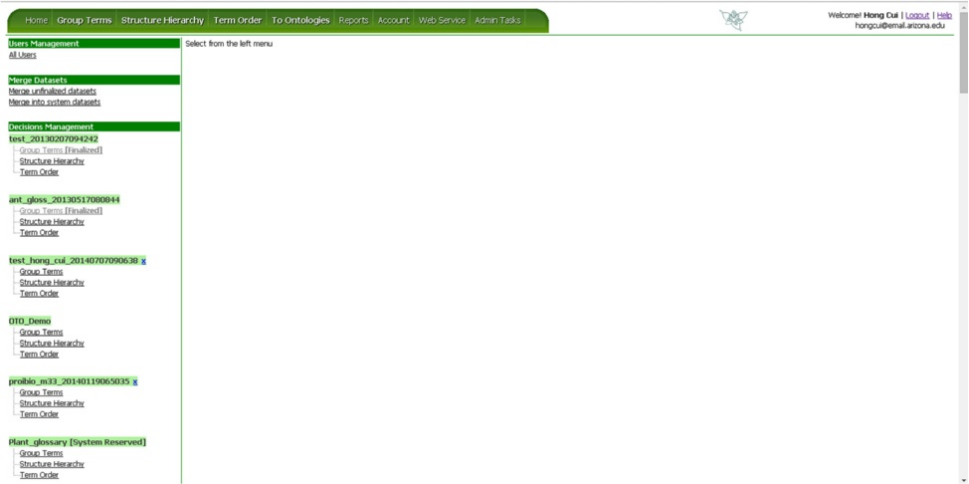
**OTO Admin Tasks page.** Administrators can manage users, merge datasets, and finalize term organization decisions on this page.

The above described functionalities are available to any user to organize a term set. Next we will introduce the administrative functions that only term set owners and administrators can access. Figure 13 provides an overview of administrative functions on the Admin Tasks page. User Management is available only to the administrators, where user accounts on OTO are approved or revoked. Decision Management allows a dataset owner or an administrator to finalize a dataset by selectively approving categorization, structure hierarchy, and term order decisions made by users. Merge datasets supports two operations: (a) unfinalized datasets can be merged by an owner or an administrator for more efficient management using the “merge unfinalized datasets” function, and (b) finalized datasets can be merged into system reserved glossaries of the same group in order to build more comprehensive glossaries. The latter can only be done by an administrator.

The Admin Tasks page also allows dataset owners or administrators to click on the name of a dataset to view the term organization process of the dataset and to click on the “x” next to a dataset to delete a dataset. When a dataset is deleted, term sets associated with all three term organization tasks and all decisions made are permanently removed.

### Dataset finalization and reopen

**Figure 14:**
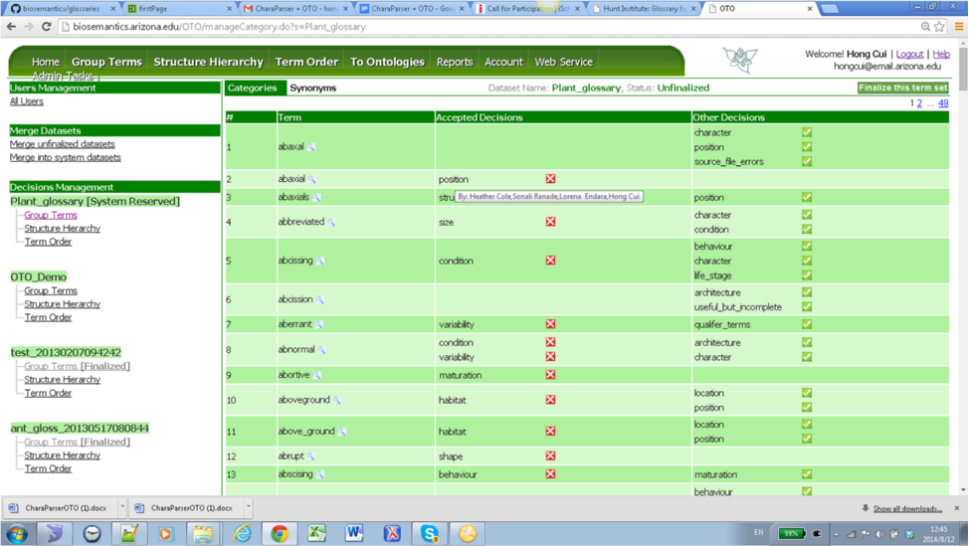
**Finalizing term categorization for a term set.** Hovering over a decision, OTO shows the users who shared the same decision. Here, four users agree that *abaxial* is_a *position*.

Clicking on any term set in any dataset in the Admin Tasks page starts the term set finalization process. Figure 14 shows the user interface for finalizing term categorizations for the dataset Plant_fna_foc. Here the reviewer (owner/admin) approves a categorization decision by clicking on the green check icon next to the category, which moves the category from the “Other Decisions” column to the “Accepted Decisions” (a click on the red “x” icon does the opposite). The reviewer learns who is responsible for a category by hovering the mouse over the category. The “Approve all System Categories” button moves the categories matching those in the related system reserved glossary into the “Accepted Decisions” column all at once. In addition to approving the categorization results, the reviewer should also approve the synonyms in a similar fashion. Not all the terms need an accepted decision before the “Finalize this dataset” button is pressed. Only the terms with an accepted decision will be saved in the final results for output, yet all information remains in the backend database. It is worth mentioning that OTO maintains a global data dictionary for triples of < term, category, taxon group > and each triple is associated with a permanent UUID [24]. These IDs are included in the results generated by the finalizing step and may be referenced by other applications (such as an ontology editor) using the terms.

**Figure 15:**
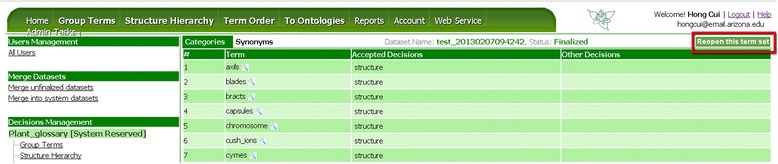
**Reopen a finalized term set.** Reopen a term set makes it editable, making it possible for an administrator to adjust accepted decisions for the terms in the term set.

Term sets for Structure Hierarchy and Term Order tasks need to be finalized in a similar fashion one by one for the results to become available for download (see Output Functionality). While finalizing a term set makes it uneditable, a finalized term set can be reopened by an owner or an admin by clicking on the name of term set in Admin Tasks, then press the “Reopen this term set” button (Figure 15). When a term set is finalized again, OTO outputs a new version of the term set on Github.

### Dataset management: merge datasets

#### Merge unfinalized datasets

**Figure 16:**
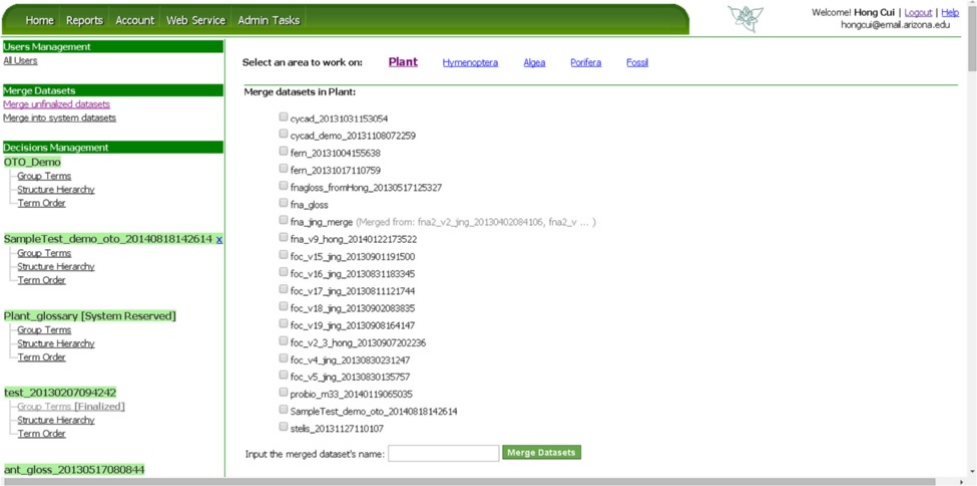
**Merge unfinalized datasets.** Datasets are organized by their taxon groups. When “Merge unfinalized datasets” is clicked, OTO displays available taxon groups. When user selects a taxon group, OTO then displays all datasets for the taxon group for the user to select and merge.

Datasets that are undergoing an organization process can be merged for more efficient management (e.g., duplicated terms are removed in the merged term set) by the owner or an admin. When “Merge unfinalized datasets” is clicked, the user is presented with a set of datasets that can be merged (Figure 16) and asked to enter a name for the merged dataset. Although OTO records the source datasets used to create a merged dataset, when a set of source datasets are successfully merged, they are permanently removed to avoid storing redundant information in the system.

#### Merge finalized term set into system term sets

**Figure 17:**
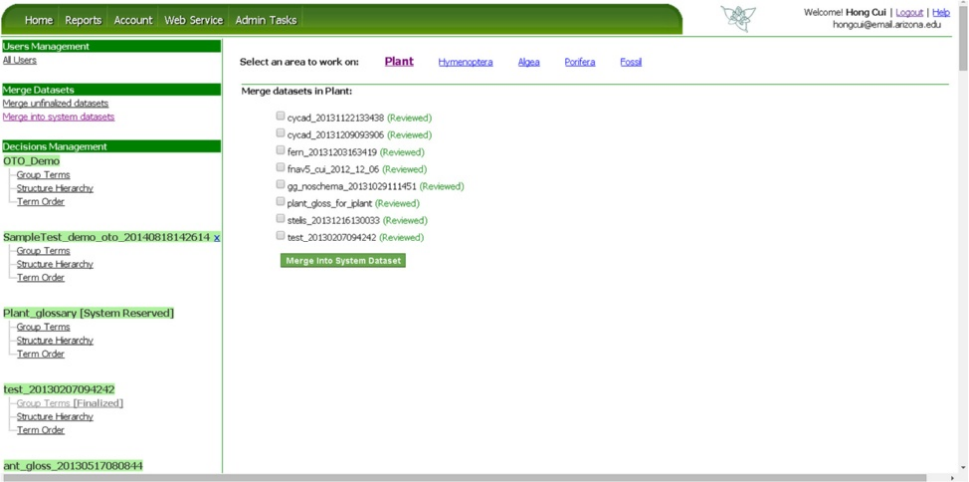
**Merge into system datasets.** Datasets are organized by their taxon groups. When “Merge into system datasets” is clicked, OTO displays available taxon groups. When user selects a taxon group, OTO then displays all datasets for the taxon group for the user to select and merge.

The mechanism OTO uses to grow system reserved datasets is through an admin merging finalized datasets into system reserved ones (Figure 17). After a merge is successfully completed, the source datasets will be deleted from OTO (but not from Github) and the new system reserved dataset will be automatically re-finalized and a new version generated on Github.

### Output functionality

**Figure 18:**
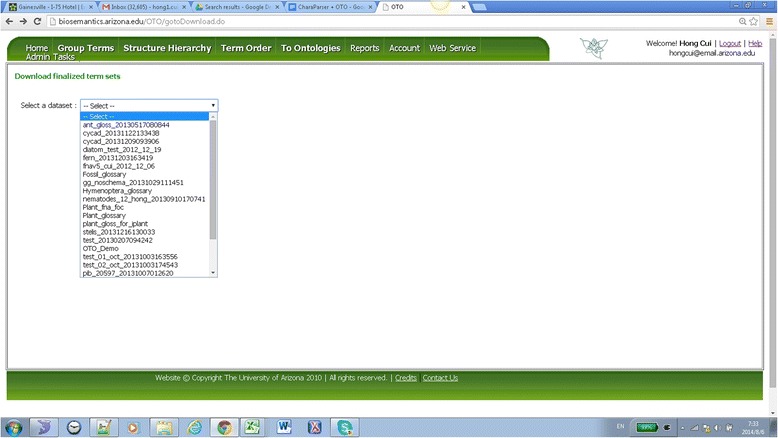
**OTO Download Dataset page.** All finalized datasets are listed in the drop-down list and user can select one to download.

During the time a dataset is being finalized and afterwards, all the decisions are frozen so that no further changes can be made to the dataset. The finalized results from Group Term, Term Order, and Structure Hierarchy are downloadable from the OTO website as SQL dump^b^ and the Group Term results also available as csv files (Figure 18). In addition, Group Term results are also output to the Github glossary repository (master branch) and each result consists of two files: *termsename*_term_category and *termsetname*_syns.

The term_category file contains all the is_a relationships between the terms and the categories. The syns file stores the synonyms. Note that synonyms are not included in the term_category file. The term_category file contains the following columns: term (string), category (string), hasSyn (1 or 0, 1 means the term has synonyms, 0 means no synonym in the syn file), sourceDataset (string, the source documents where the term is found), and termID (string, the UUID that is associated with the term/category pair). The syns file contains four columns: term (string), category (category of the term, string), termID (string, the UUID that is associated with the term/category pair), and synonym (string, the synonym). One term/category pair with multiple synonyms will result in multiple rows in the file. Note that a UUID is used to identify a ‘concept’ represented by a term/category pair, and not used to identify any synonym. In addition, both csv files contains comment lines that start with an “*” and hold metadata information about the file, such as its version number, release date, and the names of the persons who participated in the term categorization process, etc. Lastly, towards the end of the term_categorization files, we also included the natural language definitions of the categories.

### Toward ontologies

**Figure 19:**
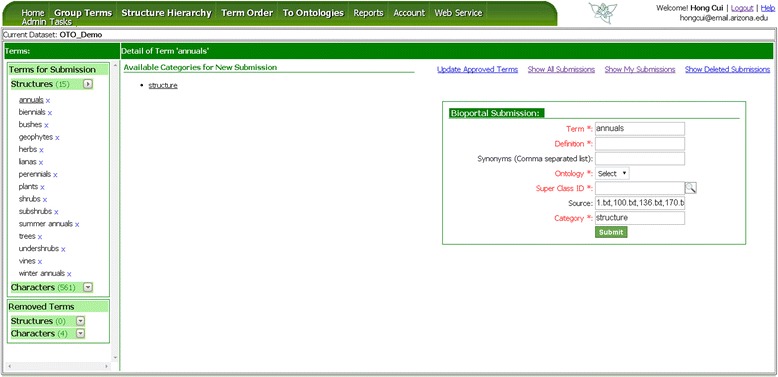
**OTO to Ontologies page.** User selects a term in the Terms column (Left), OTO provides local category for the term (Middle), and the user then fills out the term submission format (Right) to submit a term request.

Besides outputting csv files to Github, OTO is connected to ontology term trackers hosted on the NCBI BioPortal via the BioPortal REST Services (Figure 19). Users with a BioPortal id can submit terms to a set of ontologies, currently including the ontologies that roughly correspond to the system reserved glossary groups, i.e., Plant Ontology (PO), Phenotypic Quality Ontology (PATO), Hymenopetera Anatomy Ontology (HAO) and Porifero Ontology (PORO). In order to use this feature, users need to provide OTO with their BioPortal id on the Account page (Figure 3).

Terms that are submitted to ontologies through the BioPortal web service are assigned a unique temporary ID by BioPortal upon submission and will be assigned a permanent ID if the submission is accepted by the ontology. These IDs are linked to the termID (UUID) OTO generated for each approved term categorization. Although OTO holds source sentences for each term, the BioPortal web service does not take them, so they are not included in the term submission form (Figure 19).

### OTO Web services

OTO provides REST-compliant Web Services which support the functionalities of getting the available glossary types (i.e., taxon groups) in OTO, getting the most recent version of a glossary (categories and synonyms) for a certain group (Plant, etc.), getting the glossary categories and their definitions, and getting term information (categories, definitions, glossary group) associated with a term. Web services have also been implemented to support importing new term sets for various tasks. Detailed instructions can be found on the Web Service page on OTO.

### Use and benefits

OTO was initially developed to be used with CharaParser to aid semantic markup of morphological descriptions and generation of taxon-character matrices from textual descriptions. CharaParser automatically extracts domain terms from textual descriptions and uploads terms directly to OTO where the terms are reviewed and categorized by the experts. Coming full circle, the finalized term set is automatically download by CharaParser and used to generate the final markup. We have been using the CharaParser-OTO combination in projects and it has proven to be a successful strategy. In this section we describe how OTO has been used to develop a rather comprehensive plant glossary incrementally (i.e., the system reserved Plant_glossary).

We started with the FNA Categorical Glossary (FNACG, [2]), which contained 2673 concepts with categories, definitions, and synonyms defined. Here we define *concept* as a term-category pair, for example, *sweet* (taste) and *sweet* (smell) are two different concepts. By using the FNACG with CharaParser to markup the morphological descriptions in FNA v.19, then using OTO to review the extracted terms, we discovered 830 new useful concepts. Interestingly, some categorizations of FNACG did not match how the terms were used in the source descriptions. For example, the shape of leaf margins are often described as *entire*, *dentate*, *toothed*, or *lobed* in description text, but in the glossary, the category for *entire, dentate,* and *toothed* is *margin* while the category for *lobed* is *plane shape* or *solid shape*. Putting terms that are alternative values for the same attribute (margin shape) into different categories artificially increases the semantic distance among these terms. The OTO Group Term page provides useful tools to detect these kinds of issues. The Glossaries panel allows the user to see if a term is included in the existing glossary and how it is categorized. The Context tab allows the user to see all the sentences a term appears in throughout all of the source documents. Thus, based on the presented knowledge of the term’s usage in reality, the user is better informed in determining a category for the term. In the example above, we decided to merge *plane shape* and *solid shape* to one *shape* category because in descriptions the 2-D and 3-D shape terms are often used in a mixed fashion. As a result, we moved shape terms (e.g., entire, dentate, toothed) from *margin* to the *shape* category. When terms have multiple meanings/senses a user can decide to make a copy allowing one term to be put into multiple categories.

With multiple users’ effort organizing the terms in the datasets, the Plant_glossary v0.1 was released with 3293 terms and replaced the initial version FNAGC. It was then input into CharaParser to extract additional plant terms from another thirty published volumes of FNA [1] and Flora of China (FoC [25]) (FNA vols 3-5, 7-8, 19-23, and 26-27; FoC vols 4-14, 18, and 20-25). This resulted in 30 term sets or a total of 14,212 terms. Using the “Merge unfinalized datasets” function, we merged the 30 term sets into one (called Plant_fna_foc), and reduced the terms to be categorized to approximately 6000. While merging reduces the number of terms to be categorized, it keeps all the source sentences (720,000+ sentences) from all the volumes intact to provide the users with complete context information for term categorization. We used the “Copy System Decisions” function to bring the categorizations from Plant_glossary v0.1 to the current term set to promote categorization consistency, but during the categorization process, these terms were reviewed again against the new source sentences for their applicability. A set of new categories were created and some existing categories were left empty and effectively ignored. After Plant_fna_foc was finalized, it was merged into the system reserved glossary to release Plant_glossary v0.19 on Github. Table 1 summarizes the key points in the creation of Plant_glossary0.1 and 0.19

**Table 1:** **The activities related to the categorization of the terms for Plant_glossary v0.1 and Plant_fna_foc term sets**

**Term sets**	**# of unique terms**	**# of users**	**# of categorizations**	**# of conflict categorizations**	**# of comments posted**	**# of concepts**	**# of terms with multiple categories**
Plant_glossary v0.1	3293	7	11781	5295	492	3559	243
Plant_fna_foc	5776	6	10329	7107	705	6244	453
merged (Plant_glossary v0.19)	8742					9365	596

.

Table 1 shows that there were 3293 unique terms to be categorized for Plant_glossary v0.1. Seven users posted 492 comments and made a total of 11781 categorizations on these terms and 44.9% (=5295/11781) of the categorizations were in conflict with some other users. After the term set had been finalized, 243 (out of 3293) terms ended up with multiple categories and the output term set contained 3559 unique concepts. The data on Plant_fna_foc suggests a similar pattern, that is, a significant portion of terms have different categorizations by different users. These differences were largely resolved in the end (a much smaller portion of terms have multiple categories in the finalized term sets). From users’ feedback, we know that the term report (comments) was a useful tool for the user, but our experience also showed that virtual and in-person meetings can help to resolve a lot of differences as well.

No formal usability test has been conducted to quantify the user-friendliness of OTO, however, the process of reviewing and finalizing these and other datasets generated numerous constructive suggestions by the users and resulted in many feature enhancements to OTO. User feedback we received suggests that the tool has become intuitive and efficient to use, especially the Group Terms functions as they are the most used in OTO to date. There are 56 registered users and 44 datasets related to plants, algae, and invertebrates (nematodes, porifera, hymenoptera, etc.) currently on OTO.

### Future development plan

Our future development plan includes (1) Support for flexible ontology selection and use, for example, using a user specified anatomy ontology to initialize the Structure Hierarchy page. (2) Support other output formats, for example SKOS [26] or semantic wiki pages. (3) New web services for importing terms. We also plan to use Structure Hierarchy and Term Order functions to further organize FNA and FoC datasets and enhance their features based on user requests. In addition, we are working to make the term organization results available in a more user-friendly environment such as a wiki to encourage community involvement. We have created FloraTerms in TWDG’s terms wiki and made initial steps at http://terms.tdwg.org/wiki/FloraTerms.

### Related software

The key features that separate OTO from other existing thesaurus [27-31] and ontology editors [15,16] are the usage-informed consensus building features, including access to source sentences, access to the decisions and comments made by other users, and visual cues signaling disagreements. Other differences are summarized as follows: OTO is not an ontology editor and it does not deal with the syntax of formal ontologies, but it supports the most fundamental ontological relations is_a and part_of in a user-friendly manner. Library-oriented controlled vocabulary construction software [27-30] relate terms using hierarchical (denoted by BT/NT or broader term/narrower term), associative (denoted by RT, or related term), and equivalence relationships (denoted by Use or Used For), without differentiating different types of hierarchical (i.e., instance, class/subclass, part_of) or associative (i.e., developed_from, created_by, etc.) relationships. OTO separates is_a and part_of relationships clearly. Existing tools require the user to type the terms in one by one, while OTO encourages manual or software batch imports, or the use of web services (future development).

A commercial platform that integrates text mining techniques with vocabulary control and information/knowledge organization is PoolParty [31]. It enhances traditional thesaurus/taxonomy construction functions with text mining techniques and Semantic Web oriented features such as using the thesaurus/taxonomy to annotate enterprise documents and serve the annotated information as Linked Data. OTO is open source and much simpler to use as it is rather focused on consensus-based term organization.

Another tool that invites domain experts’ input to an existing ontology is NeuroLex [14], a semantic wiki for the neuroscience community and domain experts. “Essentially, NeuroLex is a place to accommodate the concepts and entities that are found in literatures and other legitimate sources that are not yet been realized within a formal ontology relevant to Neuroscience. NeuroLex allows a neuroscientist to add a new concept without having to worry about its deep semantic consequence due to incompleteness or partial truth about an asserted.” [14]. Although the Wiki platform makes it possible for the users to track the editing history of a term and take part in any discussion, promoting consensus among domain experts is evidently not the primary concern of NeuroLex.

## Conclusions

We have developed OTO to address the requirement for a user-friendly and non-technical tool to allow multiple domain experts to work collaboratively toward the creation of a controlled vocabulary that reflects the term usages in the source documents. OTO can be used to organize terms for any domain where is_a, part_of, and order relationships among terms are important for knowledge modeling and it is available online free of charge. It is open source so adapting it for other applications is possible and encouraged. Domain experts that have used OTO find the tool easy to use and have created several non-trivial glossaries which are used in ongoing projects. In the near future, we plan to release a version to the public in a wiki format to encourage further review and addition of terms and their respective definitions and illustrations.

## Availability and requirements

**Project Name**: OTO

**Project Home Page**: Source Code is available at: https://github.com/biosemantics/oto. The OTO web application is running at http://biosemantics.arizona.edu/OTO/. A video introduction to OTO can be found at http://biosemantics.arizona.edu/OTO/demo.do. Short demos that show how to use OTO can be found in the *Help* section of OTO website after login. To test the system, use username *OTOdemo* and password *OTOdemopass* to login.

**Operating Systems of Server**: Linux, Windows

**Programming Language**: JSP, JavaScript

**Other Requirements**: Java JDK 6.0 or higher, MySQL Server 5.0 or higher, Apache Tomcat 5.0 or higher.

**License**: Open source, Apache License, Version 2.0

**Restrictions to use by non-academics**: None

## Endnotes

^a^CharaParser [19] employs a bootstrap-based unsupervised machine learning method to categorize terms appearing in morphological descriptions into structure, character, and other groups. While the method does not require any training examples, it has an assumption that description sentences start with structure names and followed by characters, for example, “stems [structure] generally purple [character]”. Depending on how well the input text conforms to this assumption, in our evaluation on three real-world description collections, the accuracy of identifying structure and character terms ranged from 50% to over 80%.

^b^The SQL dump contains the tables that hold the finalized results from Group Term, Term Order, and Structure Hierarchy tasks. The tables for Group Term results have the same structure as the csv files described in the paper. The table for Term Order result currently contains seven columns: orderID (varchar, not unique), orderName (varchar, user-provided name for an order), term (varchar, a term in the order), distance (int, position counted from the beginning of the order, starting from 0), accepted (tinyint, 1), userid (int, id of the user who made the decision on this term), confirmDate (datetime, time when the order is approved). The table structure makes it easy to find a term’s position in an order, but it also means that it takes N rows to make up an order, where N = the number of terms in the order. The table for Structure Hierarchy is call “paths” and contains five columns: term (varchar, the term), pathWithName (varchar, the path of the root to the term, for example “plant-flower-stamen-anther”), accepted(tinyint, 1), userid (int, id of the user who made the decision on this term), confirmDate (datetime, time when the order is approved). As the Term Order and Structure Hierarchy functions are used more often by more users, the table structures of the results may change based on user needs.
